# Erratum to: Black phosphorus nanosheets and paclitaxel encapsulated hydrogel for synergistic photothermal-chemotherapy

**DOI:** 10.1515/nanoph-2023-0553

**Published:** 2023-09-13

**Authors:** Shoushan Sang, Zhipeng Jiang, Ning Xie, Huaxin Rao, Kedan Liao, Qinqin Hu, Ziyong Zhang, Rui Guo, Taojian Fan, Kaixian Deng

**Affiliations:** Department of Material Science and Engineering, Jinan University, Guangzhou 510632, China; Engineering Research Center of Artificial Organs and Materials, Ministry of Education, Guangzhou 510632, China; Department of Gastrointestinal Surgery, The Sixth Affiliated Hospital of Sun Yat-sen University, Guangdong Institute of Gastroenterology, Guangdong Provincial Key Laboratory of Colorectal and Pelvic Floor Diseases, Supported by National Key Clinical Discipline, Guangzhou 510655, China; Shunde Hospital, Southern Medical University (The First People’s Hospital of Shunde), Foshan 528308, China; Shenzhen Engineering Laboratory of Phosphorene and Optoelectronics, SZU-NUS Collaborative Innovation Center for Optoelectronic Science & Technology, International Collaborative Laboratory of 2D Materials for Optoelectronics Science and Technology of Ministry of Education, College of Physics and Optoelectronic Engineering, Shenzhen University, Shenzhen 518060, China

The authors regret that the original version of our paper [1], unfortunately, contained incorrect pictures in Figure 6. It appeared that HE images of heart tissue in the OSA/AHA/BPNS/PTX and OSA/AHA/BPNS/PTX + NIR groups in Figure 6 have been derived from the same original source, even though they were intending to have shown the results from differently performed experiments. After having consulted our original data, we realized that these errors had occurred while compiling the affected figure parts.

The correct version of Figure 6 was shown above.

**Figure 6:**
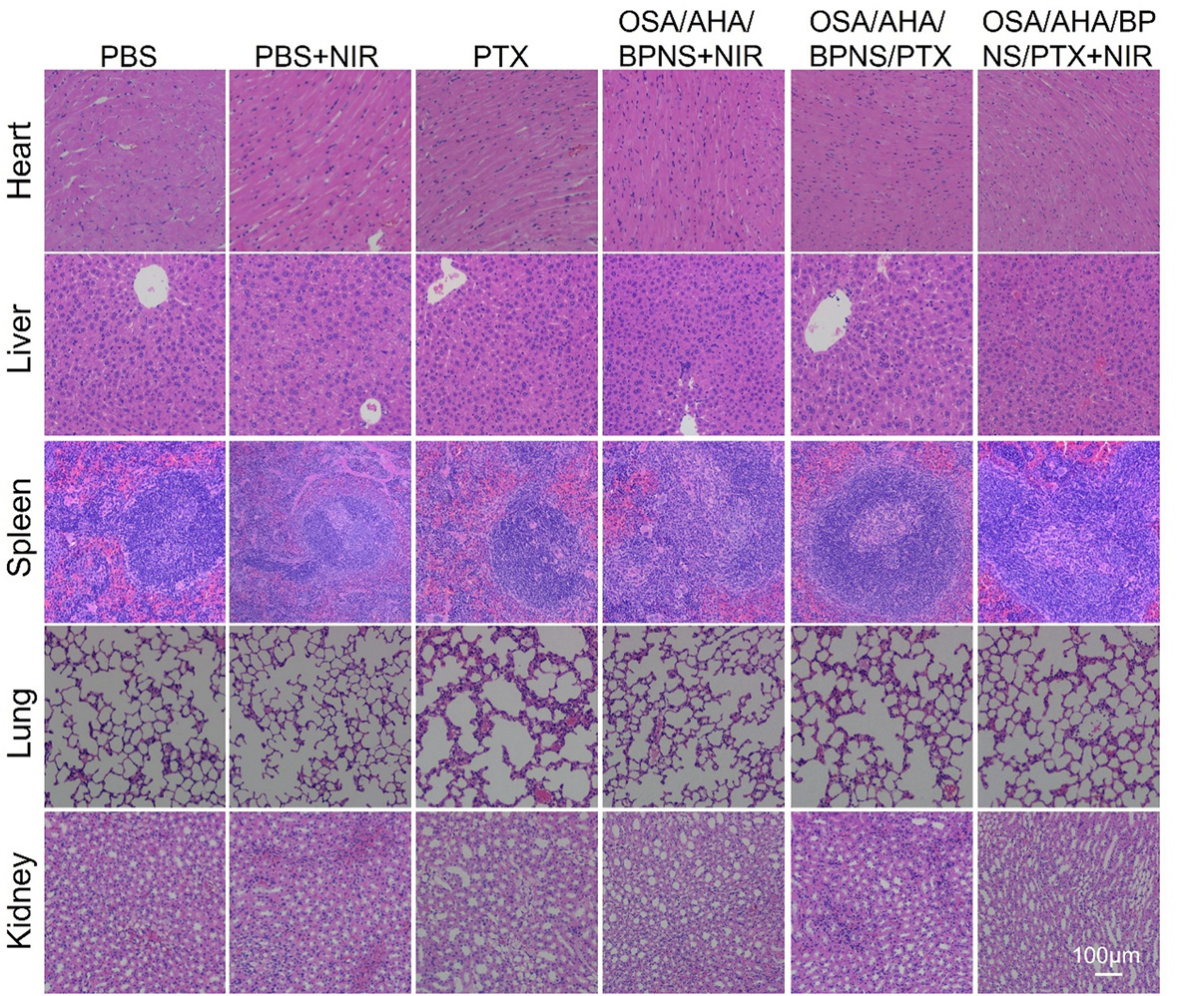
*In vivo* biocompatibility evaluation of materials, H&E staining images of mice organs underwent different treatments for 14 days. Note that 1–6 represent mice groups with different treatment.

These corrections are minor and do not alter the conclusions of the paper. The authors apologize for any inconvenience that the errors may have caused.
